# Risk and pleasure in the era of pharmacologically safe sex

**DOI:** 10.11606/s1518-8787.2024058005962

**Published:** 2024-10-11

**Authors:** Filipe Mateus Duarte, Sandra Assis Brasil, Mônica Lima, Jardel da Silva Vidal, Laio Magno, Inês Dourado, Luís Augusto Vasconcelos da Silva

**Affiliations:** I Federal University of Bahia. Postgraduate Program in Public Health. Salvador, BA, Brazil Federal University of Bahia Postgraduate Program in Public Health Salvador BA Brazil; II University of the State of Bahia. Department of Life Sciences. Salvador, BA, Brazil University of the State of Bahia Department of Life Sciences Salvador BA Brazil; III Federal University of Bahia. Institute of Psychology. Salvador, BA, Brazil Federal University of Bahia Institute of Psychology Salvador BA Brazil; IV Federal University of Bahia. Institute of Collective Health. Salvador, BA, Brazil Federal University of Bahia Institute of Collective Health Salvador BA Brazil; V Federal University of Bahia. Institute of Humanities, Arts, and Sciences Professor Milton Santos. Salvador, BA, Brazil Federal University of Bahia Institute of Humanities, Arts, and Sciences Professor Milton Santos Salvador BA Brazil

**Keywords:** HIV, PrEP, Undetectable, Young men, Biotechnologies

## Abstract

**OBJECTIVE:**

This article discusses how Pre-Exposure Prophylaxis (PrEP) and the undetectable viral load=untransmissible (UVL=U) have produced reconfigurations in the contexts of affective-sexual encounters of young gay men/men who have sex with men (MSM) living with HIV (YLHIV).

**METHODS:**

In-depth interviews were conducted with nine YLHIV, aged 18 to 29, from two studies conducted in Salvador, Bahia, in 2019 and 2021. The narratives focused on unprecedented events in the prevention and treatment of HIV/AIDS, which have allowed experiences of greater intimacy and safety but also challenges and tensions in affective-sexual relationships.

**RESULTS:**

Different moments in the experience of living with HIV reveal different narratives of YLHIV concerning the new PrEP and UVL biotechnologies. Concerns surrounding possible HIV transmission or the obligation to reveal serology are more prominent among young people with the most recent diagnosis. In contrast, those with more extended serology experience are more comfortable and confident in the face of new technologies and their significant effects on sexual encounters. However, controversies remain regarding the moral and behavioral consequences of their use. Some YLHIV re-update concerns and bring reports about the continuity of stigma toward people living with HIV. Others emphasize the benefits of biomedical advances, opening up new interactive possibilities, including without the use of condoms, highlighting the existence of other practices, knowledge, dynamics, and ways of negotiating risk/care, with tensions in the field of sexuality itself.

**CONCLUSIONS:**

We reiterate the need to resume public policies in the field of HIV/AIDS beyond biomedical strategies, highlighting vulnerabilities, the dissemination of information about new HIV prevention and treatment technologies, respect for people’s autonomy in their preventive choices, and the development of strategies to combat the stigma associated with HIV/AIDS.

## INTRODUCTION

Expanding the use of antiretroviral drugs (ARV) to prevent HIV through pre- and post-exposure prophylaxis (PrEP and PEP, respectively) and treatment as prevention (TasP) is an effective response to combating the HIV/AIDS epidemic. While having an impact on the prevention of new infections, using these pharmacological technologies brings benefits to affective-sexual relationships. It raises challenges and new questions for the daily lives of health services and people^1^^), (^^2^.

The evidence on TasP, produced by several studies around the world, is increasingly robust in arguing that people living with HIV (PLHIV) and with an undetectable viral load (UVL) do not transmit the virus in their sexual relations, which is agreed to call *undetectable equal to untransmissible* (U=U)^3^. This biotechnological advance has produced more comfort in the affective-sexual life of PLHIV, representing a marker of greater safety and alleviating concerns surrounding their sexual encounters^4^, although reports of stigma, feelings of fear/guilt, and fears of possible transmission persist^2^.

On the other hand, different studies have demonstrated the effectiveness of PrEP in preventing new HIV infections^5^^), (^^6^^), (^^7^^), (^^8^. As long as it is consistently used, PrEP works as a chemical barrier to HIV, offering high protection even in sexual relations without the physical barrier of condoms. Thus, prevention gained a new facet, highlighted by the use of ARV by people not living with the virus. This aspect has given them a new level of management, control, and maximization of sensory pleasure, allowing them to experience intimacy without fear of HIV infection, even though the risk of other sexually transmitted infections (STIs) continues to exist.

In this context of pharmacologically safe sex, the use of ARV has redefined not only the way some people deal with HIV but also their sexuality, the management of their sexual health, and the conventional conception of risk. In particular, the concept of “pharmacopower” developed by Preciado^9^ offers a valuable lens to understand this context of the profound intersection between pharmacology, corporeality, and identity. By continuing to examine what Foucault^10^ called “biopower”, i.e., the power strategies that act on life on a population scale, Preciado highlights how substances and medicines have played a crucial role in health/illness experiences, producing people’s perceptions of themselves and their relationships with others.

Thus, the concept of “protected sex” has been redefined, as the emergence of these new technologies now blurs its borders, given that prevention does not occur exclusively through condoms but also begins to be internalized in the body itself through the ingestion of chemical substances^11^. On the other hand, since PrEP and U=U stand out for providing their users with a more pleasurable sexual experience and reducing anxiety related to HIV transmission^12^^), (^^13^^), (^^14^, there are fears among healthcare professionals and even among those who use these technologies about the possibility of greater engagement in multiple sexual partnerships, infidelity, greater frequency of sexual relations and reduction or even abandonment of condoms^15^^)-(^^18^. At the same time, the reproduction of negative images persists concerning cis gay/homosexual men, seen as “excessive” subjects, or, as Kane Race^19^ problematizes, a certain “dread” of the resumption of “unrestrained” sex between men.

In this direction, we seek to understand how these new HIV prevention and treatment technologies have mediated or reconfigured sex among young cis gay men living with HIV (YLHIV). More precisely, we highlight how this aspect appears in the context of the different temporalities of the diagnosis of these young people, considering both the most recent period of experience with serology and the more extended period. Thus, from the perspective of YLHIV, we will highlight both the changes, ambivalences, and tensions that persist in affective-sexual encounters, especially distrust and controversies emerging from the introduction of pharmacological technologies, such as PrEP and UVL=U.

## METHODS

We started from two different studies (*PrEP15-19* and *Sociabilidades Positivas* (S+)) in Salvador, Bahia. We conducted in-depth interviews with nine young gay men/men who have sex with men (MSM) living with HIV, based on a semi-structured script, which covered topics such as treatment and care routines, engagement in affective-sexual relationships, and their post-diagnosis perspectives of HIV.

The first round of interviews took place at the end of 2019, between October and December, with four young people between 18 and 19 years old during the *PrEP15-19* study. More methodological information about this study can be identified in Dourado et al.^20^ and Magno et al.^21^. These young people were diagnosed with HIV during their first visits to the combined prevention clinic where they initially went to seek PrEP. After diagnosis, they received guidance on prevention and healthcare and were referred for treatment at the SUS. Subsequently, they were invited by a healthcare professional from the clinic to give an interview to the study’s qualitative research team. The interviews occurred between 10 days and three months after diagnosis. These young people were starting ARV treatment.

The second interview sequence took place approximately one year after the first round, in January 2021, with five other young people aged between 22 and 29. These participants participated in the S+ study, and their team of researchers has followed their trajectories since 2016, the year of their diagnoses^2^. At the time of the interview, these young people reported having an undetectable viral load=untransmissible (UVL=U). Throughout the text, we will quote fragments of the interviewees’ narratives in quotation marks, followed by their fictitious names, as part of our ethical commitment to anonymity.

A team of researchers trained in qualitative research in the health field conducted the interviews. They were carried out individually, in person, in a private room on the premises of the *PrEP15-19* study clinic. Although produced within the scope of two different studies, we chose to bring together the set of materials from both, as we understand that they outline dialogical aspects about the affective-sexual trajectories of the young participants.

The analysis of the materials produced began through exploratory readings to identify themes and questions that moved in the narratives around the serological condition and the contexts of affective-sexual encounters of these young people. Subsequently, the common aspects of these narratives were qualified, with emphasis on new developments in the HIV/AIDS epidemic, such as the advent of PrEP and UVL=U. Thus, throughout the analysis, three aspects or themes highlighted by the materials are discussed: (1) the mismatch between pharmaceutical and medical advances, which demarcate greater safety concerning HIV infection/transmission, and the daily experience of serology still marked by stigma and fear of rejection; (2) the reconfiguration of how YLHIV deal with serology and everyday situations of affective-sexual encounters, especially throughout living with HIV and with access to evidence about their health condition; and, finally, (3) the reproduction of old moral dilemmas linked to the exercise of sexualities.

It is understood that the “personal” narratives of these YLHIV connect to the more generalized plot about the current HIV/AIDS epidemic. At the same time, it is possible to recognize that different stories are re-updated, emerge, and coexist concerning this plot^22^. Therefore, throughout the analysis, the importance and strength of narratives are considered, with their heterogeneity and polyphony, opening new questions for this field of studies^23^.

Regarding the participants’ characterization, seven interviewees identified themselves as Black (Black and Mixed-race) and two as White (Chart 1). At the time of the interview, all four participants in the *PrEP15-19* study reported living with their parents. Among the five participants in the S+ study, two reported living with their parents, two alone, and one shared a home with friends. Regarding clinical characterization, all were asymptomatic at the time of the interview.

**Chart 1:** Characterization of participants

Fictitious name	Age^a^	Sexual orientation	Race/color	Education	Relationship	Time since HIV diagnosis^a^
**PrEP15-19 Study**						
Caio	18	Gay	Mixed-race	Incomplete Secondary Education	Single	10 days
Ruan	18	Gay	Mixed-race	Incomplete Secondary Education	Single	1 month
Henrique	19	Bisexual	Black	Incomplete Secondary Education	Single	3 months
Eduardo	19	Gay	Mixed-race	Incomplete Secondary Education	Single	5 months
**Sociabilidades Positivas Study**						
Samuel	22	Gay	Black	Incomplete Higher Education	Single	4 years
Bernardo	27	Gay	Mixed-race	Complete Higher Education	Single	4 years
Caetano	29	Gay	White	Complete Higher Education	Dating	4 years
Douglas	27	Gay	White	Complete Secondary Education	Dating	4 years
Ramon	24	Gay	Mixed-race	Complete Secondary Education	Single	4 years

Source: Prepared by the authors. ^a^ At the time of the interview.

Taking their ages at the time of the interview as a reference, these young people were between 18 and 24 years old when diagnosed, showing that these are infections that occurred at the beginning of their sexual experiences. This element supports the data presented by the Epidemiological Bulletins of recent years, which have demonstrated the resurgence of the epidemic in the youth age segment and the need to expand the debate on socially configured susceptibilities, which produce vulnerabilities to HIV/AIDS, mainly due to the unequal availability of resources for people to protect themselves and to produce health/care.

The *Sociabilidades Positivas* study was approved by the Ethics Committee (IRB/IEC) of the Institute of Collective Health at the Federal University of Bahia (ISC/UFBA) (No. 1,684,862/2016). The *PrEP15-19* study was approved by the IRBs/IECs of the World Health Organization (Identification: *Fiotec-PrEP Adolescent study*) and ISC/UFBA (No. 3,224,384). The interviews were carried out following the guidelines of Resolutions 466/2012 and 510/2016 of the National Health Council on ethics in research in human and social sciences in health.

## RESULTS AND DISCUSSION

As we enter the fifth decade of the HIV/AIDS epidemic, centered on progress in drug-medical treatment and prevention, new relational arrangements emerge from the use of drug-technology. In this sense, the following stand out: affective-sexual relationships between serodifferent people, in which the undetectable status of one of the partners is a marker of prevention within the relationship or relationships in which one partner is undetectable, and the other is using PrEP; or even a sense of companionship between “similars”, characterized by the “preference” of PLHIV for relating to partners of the same serological status, thus removing specific dilemmas, tensions, and questions about their serology^2^.

At the time of these unprecedented events in the history of the epidemic, challenges persist among PLHIV when considering whether they should reveal their serology to their partners, for fear of being stigmatized and rejected and, in turn, the sense of personal responsibility in avoiding a possible infection/transmission of the virus. Such aspects are linked, for example, to the idea that HIV is a condition that should be ashamed of, as it is mainly linked to “deviant” sexual practices. This framework allowed Treichler^24^ to coin, from the first decade of the epidemic, the expression “epidemic of meanings”, which became classic when analyzing how language - not just medical and scientific - produces what we conceive as HIV/AIDS, providing the basis for the production of stigmas and their practical effect, which is discrimination.

In this study, it is argued that this framework is updated in the face of other actors/actants, such as UVL=U and PrEP^18^, which, being part of a network of interactions, have contributed to reconfiguring or giving unprecedented contours not only to the clinical practice of prevention and treatment but also affective-sexual relationships. This is the case, for example, of “living with HIV” and, however, claiming to be “negative using PrEP”, as highlighted by one of the interlocutors, with UVL=U, based on his forays into relationship apps:

I see many people who have HIV/AIDS and say they are using PrEP. I see this in apps, conversations, and groups. In one group, they said someone had AIDS, and he said he was taking PrEP and was negative. I spoke to a guy who told me he was undetectable HIV+; I wanted to leave him hanging, but I couldn’t, so I said I was negative on PrEP. (Bernardo, 27 years old)

This narrative reiterates how the daily experience of serology still seems out of step with the drug-medical advances that occurred in the last years of the epidemic. In other words, even though these new advances may blur the border between “negative” and “positive”, or instead, between “absence” and “presence” of the HIV virus^25^, considering the undetectable category here, the persistence of fear or concern of being associated with HIV/AIDS must be highlighted, with consequent discrimination. In this sense, in addition to the medicalization and normalization of HIV^26^^), (^^27^ as a chronic and treatable disease, one cannot forget difficulties and dilemmas that persist in this historical moment that Simões calls the “new AIDS”^28^, resulting from successful biomedical interventions on its clinical and epidemiological reality.

It is worth emphasizing that different scholars on the theme^29^^), (^^30^^), (^^31^ have reiterated that, currently, there is an emphasis on individual perspectives in confronting the HIV/AIDS epidemic, with the relative displacement of prevention from community/collective spaces to the interior of the medical clinic, primarily focused on the act of medicating. In this sense, a set of new knowledge about HIV and new technologies, such as U=U and PrEP, has not circulated beyond specific niches, contributing to maintaining the persistent nature of stigma. Despite this, as discussed in this article, there are also essential changes made possible by these new biotechnologies. In the following sections, some of these aspects of living with HIV and affective-sexual relationships take on different contours based on different temporalities.

### The time of serological discovery: ruptures and tensions in focus

Positive HIV serology continues to create dilemmas and feelings of guilt, individualizing, simplifying, and minimizing other elements surrounding this health condition^32^^), (^^15^. As with other chronic diseases concerning biographical ruptures^33^-but mainly due to the stigma of HIV, linked to the idea of “promiscuous sexualities”^26^ and “dangerous bodies”^34^^), (^^35^-this new diagnosis also raises questions about the subjects themselves and their ways of life^15^. Thus, re-engagement in relationships can imply difficulties, especially at the beginning of the experience of serology, as can be exemplified by reports from some of the interlocutors with a more recent time since diagnosis.

The first time I had [sex] after the diagnosis, I was scared. But I tried to calm down, I tried not to think. There were a few moments when I thought about the issue of HIV and everything else, afraid of so many things happening, of the condom breaking and this or that happening. [...] I feel a little like that... I just analyze each step because anything someone does could end up affecting me, and I’ll have to end up telling someone [that I have HIV], you know?. (Caio, 18 years old)

Regarding the dilemma of serological disclosure-“have to end up telling”-Agostini et al.^36^ emphasized in their study how the management of the seropositivity condition presents challenges in the context of affective-sexual relationships, including the need to establish a bond of confidence before the topic of serology is addressed. Thus, secrecy management plays a vital role in these relationships and is maintained as a protection strategy against stigma, emotional rejection, and discrimination. The authors also discuss how some of these young people show great concern about avoiding transmission and adopting prevention strategies, such as condoms.

Even though tensions related to sexual involvement are not limited to the initial period of experiencing serology, the narratives of the interlocutors of the *PrEP15-19* study-as reported above by Caio-emphasize this aspect more than the narratives of the young people in the S+ study. Regarding this, from a perspective of meaning construction over time, Anjos^37^ observed that the initial shock of the diagnosis often progresses to a gradual acceptance of this condition by YLHIV. The author explored how living with HIV is permeated by an initial period of emotional adjustment and difficulties in revealing serological status to family members and partners due to the concern of being stigmatized and excluded. However, he highlights the resilience and search for a meaningful life on the part of these young people despite the challenges, fears, and uncertainties present in living with HIV. He also emphasizes how the temporality of the diagnosis, whether recent or longer, plays a significant role in the way YLHIV live with their serology, adapt to this health condition, and deal with their affective-sexual encounters.

While it is essential to recognize that stigma, fear, and discrimination are still significant obstacles regardless of how long since diagnosis, these studies signal that those living with HIV for a more extended period often have a better understanding of their health condition, develop resilient coping strategies, learn to manage practical and emotional aspects of serology and establish a more stable sense of positive identity concerning their positive HIV status.

Newly diagnosed interlocutors and those still at the beginning of treatment highlighted stories about how post-diagnosis sexual involvement is surrounded by tensions, which can mean a greater frequency of concerns that seemed less important before the diagnosis, such as condom use. This care is related to the new sense of risk or “threat” to others that the HIV diagnosis starts to mobilize in the lives of these young people, and condoms begin to be seen as something compulsory or even as a moral obligation.

I use condoms for everything now, condoms for everything. Not to mention... I’m being much more careful. Even with a condom, I think it’s cool to go a little deeper before doing anything. Mainly the part about using condoms for everything; it was after the project [PrEP15-19, in which he was diagnosed]. I didn’t do that, no. (Ruan, 19 years old)

Nowadays, I always use [condoms], whether he wants to or not. If he doesn’t want to, I won’t have sex. Before, it didn’t happen like that. If he wanted, he would use it; If he didn’t want to, fine [he wouldn’t use it] (Eduardo, 19 years old)

These elements remain throughout the serology experience, implying significant challenges for YLHIV. However, at different moments in their trajectories, other concerns come to the surface, which still concern safety but introduce new meanings to sexual relations, such as freedom and pleasure.

### PrEP and UVL=U on the scene: new interactive possibilities for negotiating sex and its tensions

In the context of pharmacological sex safety, strategies such as PrEP and UVL=U have enhanced the sexual well-being of PLHIV^38^. We highlight the stories of sexual encounters reported by Caetano-29 years old and living with HIV since he was 24 years old-and Douglas-27 years old and living with HIV since he was 25 years old. These are contexts in which the use of condoms is contingent on elements such as “carelessness”, “comfort”, “stability”, “trust”, “negotiation”, and “pleasure.”

When we were arranging [a meeting], he made it clear that my HIV status made no difference to him and that he used PrEP. [...] I think it’s the first time I’ve had sex without a condom, without being too worried about the other person. [...] Now, I’m having the pleasure of having sex with someone who uses PrEP, and sometimes, we have sex without a condom. This comfort zone concerning protection makes us enjoy it more. (Caetano, 29 years old).

[...] Nowadays, we have more sex without condoms than we used to [...]. Now that I’m undetectable, he’s stopped [taking PrEP]. He even discovered a different way to use PrEP: use it for one day, then two days, then two more. (Douglas, 27 years old).

Through these fragments, we emphasize aspects that mark the current context of HIV/AIDS, i.e., the reference to new biotechnologies as elements that make a difference in decision-making within affective-sexual relationships. The narratives of YLHIV that achieved viral undetectability point to elements that mark the experience of serology today and offer a new dynamic concerning safety. In this case, undetectability produces a sense of freedom in exploring sexual pleasure and changes in the way these YLHIV enjoy relationships without excessive concerns about virus transmission while also facing challenges involving communication and negotiation with partners. In line with the reflections of Preciado^9^, this context highlights the intersection between pharmacology, corporeality, and identity, in which medicine and technology play fundamental roles in redefining sexual experiences and constructing new meanings for intimate relationships.

The experiences reported by these and other young people demonstrate how, based on availability and willingness to use technologies other than condoms, people can combine and negotiate uses, balancing sexual concerns and interests as they wish to experience pleasure in a “freer” and “barrier-free” way. As highlighted by Silva et al.^18^, such fragments show how prevention can be adjusted or reconfigured according to the performance of technologies (U=U, PrEP, condom), the type of relationship (fixed, casual, open, closed), the time of diagnosis or even in situations where the risk-pleasure relationship is discussed between partners.

However, it is necessary to highlight that, in addition to the evidence of the benefits and effectiveness of PrEP and UVL=U as HIV prevention, some of these young people also have concerns about the moral and behavioral consequences of the availability of these new preventive resources. These concerns are mainly linked to the negative image of “promiscuity” attributed to gay men^39^. As we will see later, some of our interlocutors perceive sex without a condom, even among those who are using PrEP, as an uninformed action or something “trivialized.” Furthermore, on a symbolic level, the association of PrEP with “promiscuity” and “irresponsibility” can lead to the stigmatization of people who adhere to the method, especially when it is considered a strategy linked to “madness”, “lack of protection” and to “carelessness.”

I think PrEP is important, but I think there has been a trivialization of the idea of safety after PrEP. PrEP is important, but condoms are essential [...], but then, some people think that, because they are taking PrEP, they are protected from everything. Then, on dating apps, you see that people only trust PrEP. (Samuel, 22 years old).

Many people are relying on PrEP, but PrEP does not protect against other diseases. [...] It’s just that sometimes when a person is dating, they can feel safe having sex without a condom. There are people who are crazy. There are friends of mine who have sex without a condom when they are dating. I’m on the app a lot, and I see many people wanting to go bareback up and down. People put a lot of trust in a pill that can fail, in this case, PrEP. I’ve seen documentaries of people who took PrEP and still got HIV. [...] If it’s a sloppy person, it won’t work. (Bernardo, 27 years old)

When discussing confidence in the effectiveness of the medication, these young people reveal their concerns about the use of PrEP associated with the risks of other STIs and the negative image of gay “promiscuity” on dating apps. Their suspicions concern the risks that, in this case, are seen as negative and, therefore, as something to be avoided. Lupton^40^ highlights that this emphasis on avoiding risk is strongly associated with a desire to control and rationalize life and the body, avoiding the “vicissitudes of fate.” In this scenario, sex without a condom, even among those who are undetectable or using PrEP, can take on the appearance of “recklessness” and “distrust”, being conceived more as poorly behaved sex, susceptible to “failure”, “lack of control”, or “unreason/madness”, rather than as a “balance point” between costs and benefits of risk and preventive behavior, as highlighted by Eaton and Kalichman^41^, or even as part of a complex network of negotiation of losses and gains^42^, in which fear/risk is weighed against sexual interests and the search for pleasure^14^.

For Spink^43^, although it continues to announce the possibility of loss (“getting HIV/STI”), the risk can also be experienced as a positive experience (negotiation, intimacy, pleasure) since it is possible to challenge limits, “calibrate” prudence and desire, loss and reward, danger and pleasure, and risk and safety, breaking with preventive rationality that disregards new resources and technologies that affect desire, intimacy, and safety. PrEP and UVL=U happen amid these controversies and tensions in people’s daily lives, mainly because it is in fact in these interactions, practices and concrete situations that these technologies come into existence.

## CONCLUSIONS

This article addressed YLHIV narratives, highlighting some aspects that persist in the HIV-positive experience, mainly in the context of their sexual partnerships. While moral conflicts that persist concerning HIV have been highlighted, the findings point to a scenario marked by the reconfiguration of the affective-sexual experiences of YLHIV through the new pharmacological technologies of UVL=U and PrEP, which produce a greater sense of safety and pleasure. In turn, confidence in ARV medications acting as a chemical barrier to HIV transmission/infection also raises questions about other aspects of prevention, such as access, equity, citizenship, and the right to health.

Indeed, new studies will be necessary to monitor possible changes in this scenario, considering the different moments of living with HIV, other life trajectories, and new technologies that may emerge. In this article, for example, concerns about possible HIV transmission or the obligation to reveal serology are more prominent among young people with the most recent diagnosis. In contrast, those with more extended serology experience are more comfortable and confident in the face of new technologies and their significant effects on sexual encounters, as these medications reshape experiences about themselves and relationships with others, even though controversies persist.

In this direction, the need to resume public policies in the field of HIV/AIDS that articulate evidence on new prevention and treatment technologies with mobilization experiences in which the communities most affected by the epidemic are protagonists is also highlighted. These policies must be anchored in the horizon of human rights and, in turn, be culturally sensitive, considering generational differences, different social belongings, and respect for the autonomy of preventive and care choices. Furthermore, they must develop strategies to reduce vulnerabilities by confronting material, cultural, and political contexts of social injustice and discrimination against people and communities affected by the epidemic^29^.

Finally, as Mol^44^ informs, care is multiple, as it is carried out in practices, with their unpredictability, different relationships, and conditions, and new experiments may occur to produce a possible existence. In the field of prevention, this aspect is relevant as it is necessary to consider the existence of other practices, knowledge, dynamics, and forms of risk/care negotiation, taking into account the dimensions of desire/pleasure and the tensions in the field of sexuality itself, as a “border zone” of diverse coexistence, for example, of norms and transgression^45^.

In this sense, PrEP and UVL=U raise challenges but also opportunities for health services, professionals, and users when it comes to confronting taboos related to sexuality and HIV/STI. Furthermore, they provoke the need to produce new prevention and care approaches that consider the other’s vulnerabilities and processes of stigmatization or devaluation.

## References

[1] Zucchi ME, Grangeiro A, Dulce F, Pinheiro FT, Alencar T, Ferguson L (2018). Da evidência à ação: desafios do Sistema Único de Saúde para ofertar a profilaxia pré-exposição sexual (PrEP) ao HIV às pessoas em maior vulnerabilidade. Cad Saúde Pública.

[2] Silva LAV, Duarte FM, Lima M (2020). “Eu acho que a química entrou em reprovação”: Relações afetivo-sexuais de homens jovens vivendo com HIV/aids e com carga viral indetectável. Sex., Salud Soc.

[3] Broyles LN, Luo R, Boeras D, Vojnov L (2023). The risk of sexual transmission of HIV in individuals with low-level HIV viraemia: a systematic review. Lancet.

[4] Grace D, Chown SA, Kwag M, Steinberg M, Lim E, Gilbert M (2015). Becoming “undetectable”: longitudinal narratives of gay men’s sex lives after a recent HIV diagnosis. AIDS Educ Prev.

[5] Grant RM, Lama JR, Anderson PL, McMahan V, Liu AY, Vargas L (2010). Preexposure chemoprophylaxis for HIV prevention in men who have sex with men. N Engl J Med.

[6] Baeten JM, Donnell D, Ndase P, Mugo NR, Campbell JD, Wangisi J (2012). Antiretroviral prophylaxis for HIV prevention in heterosexual men and women. N Engl J Med.

[7] Choopanya K, Martin M, Suntharasamai P, Sangkum U, Mock PA, Leethochawalit M (2013). Antiretroviral prophylaxis for HIV infection in injecting drug users in Bangkok, Thailand (the Bangkok Tenofovir Study): a randomised, double-blind, placebo-controlled phase 3 trial. Lancet.

[8] McCormack S, Dunn DT, Desai M, Dolling D, Gafos M, Gilson R (2016). Pre-exposure prophylaxis to prevent the acquisition of HIV-1 infection (PROUD): effectiveness results from the pilot phase of a pragmatic open-label randomised trial. Lancet.

[9] Preciado PB (2018). Testo Junkie: sexo, drogas e biopolítica na era farmacopornográfica.

[10] Foucault M, Foucault M (1988). História da sexualidade 1: a vontade de saber.

[11] Dean T (2015). Mediated intimacies: raw sex, Truvada, and the biopolitics of chemoprophylaxis. Sexualities.

[12] Barreto VHS (2020). Responsabilidade, consentimento e cuidado. Ética e moral nos limites da sexualidade. Sex, Salud Soc.

[13] Mabire X, Puppo C, Morel S (2019). Pleasure and PrEP: pleasure-seeking plays a role in prevention choices and could lead to PrEP initiation. Am J Mens Health.

[14] Silva-Brandao RR, Ianni AMZ (2020). Sexual desire and pleasure in the context of the HIV pre-exposure prophylaxis (PrEP). Sexualities.

[15] Krakower D, Ware N, Mitty JA, Maloney K, Mayer KH (2014). HIV providers’ perceived barriers and facilitators to implementing pre-exposure prophylaxis in care settings: a qualitative study. AIDS Behav.

[16] Calabrese SK, Underhill K (2015). How stigma surrounding the use of HIV preexposure prophylaxis undermines prevention and pleasure: a call to destigmatize “Truvada Whores”. Am J Public Health.

[17] Silva LAV da, Duarte FM, Magno L, Dourado I, Squire C (2021). Moral barriers to HIV prevention and care for gay and bisexual men: challenges in times of conservatism in Brazil. Sociol Health Illn.

[18] Silva LAV, Brasil SA, Duarte FM, Cunha LA, Castellanos MEP (2023). Between risk and pleasure: reflections on HIV prevention and care in the current context of PrEP use by men who have sex with men. Cad Saúde Pública.

[19] Race K (2015). Reluctant objects: sexual pleasure as a problem for HIV biomedical prevention. GLQ: A J of Lesbian and Gay Studies.

[20] Dourado I, Magno L, Greco DB, Zucchi EM, Ferraz DAS, Westin MR, Grangeiro A (2023). Interdisciplinarity in HIV prevention research: the experience of the PrEP1519 study protocol among adolescent MSM and TGW in Brazil. Cad Saúde Pública.

[21] Magno L, Soares F, Zucchi EM, Eustórgio M, Grangeiro A, Ferraz D (2023). Reaching out to adolescents at high risk of HIV infection in Brazil: demand creation strategies for PrEP and other HIV combination prevention methods. Arch Sex Behav.

[22] Squire C, Andrews M, Squire C, Tamboukou M (2013). Doing Narrative Research.

[23] Mol A, Law J (2004). Embodied action, enacted bodies: the example of hypoglycaemia. Body & Society.

[24] Treichler PA (1987). Aids, homophobia, and biomedical discourse: an epidemic of signification. Cultural Studies.

[25] Lee N (2013). Becoming-undetectable. E-Flux Journal.

[26] Squire C (2010). Being naturalised, being left behind: the HIV citizen in the era of treatment possibility. Critical Public Health.

[27] Flowers P, Davis M, Squire C (2010). HIV treatment and prevention technologies in international perspective.

[28] Simões J (2018). Gerações, mudanças e continuidades na experiência social da homossexualidade masculina e da epidemia de HIV-Aids. Sex., Salud Soc.

[29] Calazans GJ, Parker R, Terto V (2022). Refazendo a prevenção ao HIV na 5ª década da epidemia: lições da história social da Aids. Saúde debate.

[30] Brigeiro M, Monteiro S (2023). Pre-exposure prophylaxis for HIV in Brazil: hopes and moral panic in the social construction of a biomedical technology. Cult Health Sex.

[31] Aggleton P, Parker R (2015). Moving beyond biomedicalization in the HIV response: implications for community involvement and community leadership among men who have sex with men and transgender people. Am J Public Health.

[32] Silva LAV, Santos M, Dourado I (2015). Entre idas e vindas: histórias de homens sobre seus itinerários ao serviço de saúde para diagnóstico e tratamento de HIV/Aids. Physis.

[33] Bury M (2011). Doença crônica como ruptura biográfica. Tempus.

[34] Cunha CC (2012). Os muitos reveses de uma sexualidade soropositiva: o caso dos jovens vivendo com HIV/AIDS. Sex., Salud Soc.

[35] Cunha CC (2014). Modos de fazer sujeitos na política de Aids: a gestão de jovens vivendo com HIV/AIDS. Século XXI Revista de Ciências Sociais.

[36] Agostini R, Maksud I, Franco T (2018). “Eu tenho que te contar um negócio”: gestão da soropositividade no contexto dos relacionamentos afetivo-sexuais de jovens vivendo com HIV. Sex, Salud Soc.

[37] Anjos DF (2012). Quando três tempos se encontram: sentidos e ressignificações de jovens vivendo com HIV/Aids.

[38] Zimmermann HML, Postma LR, Achterbergh RCA (2021). The impact of pre-exposure prophylaxis on sexual well-being among men who have sex with men. Arch Sex Behav.

[39] Dubov A, Galbo P, Altice FL, Fraenkel L (2018). Stigma and shame experiences by MSM who take PrEP for HIV prevention: a qualitative study. Am J Mens Health.

[40] Lupton D (1999). Risk.

[41] Eaton LA, Kalichman SC (2007). Risk compensation in HIV prevention: implication for vaccines, microbicides, and other biomedical prevention technologies. Curr HIV/AIDS Rep.

[42] Silva LAV (2012). Redução de riscos na perspectiva dos praticantes de barebacking: possibilidades e desafios. Psicol Soc.

[43] Spink MJP (2010). Psicologia social e saúde: práticas, saberes e sentidos.

[44] Mol A (2008). The logic of care: health and the problem of patient choice.

[45] Gregori MF (2016). Prazeres perigosos: erotismo, gênero e os limites da sexualidade.

