# Supplementary material for: Risk and pleasure in the era of pharmacologically safe sex
Source: Rev Saude Publica. 2024 Oct 11;58(Suppl 1):7s. doi: 10.11606/s1518-8787.2024058005962 (PMC11573377; doi:10.11606/s1518-8787.2024058005962)
Supplement: Translation

# Translation: Risco e prazer em tempos de sexo farmacologicamente seguro

**Authors:** Filipe Mateus Duarte, Sandra Assis Brasil, Mônica Lima, Jardel da Silva Vidal, Laio Magno, Inês Dourado, Luís Augusto Vasconcelos da Silva

**Affiliations:** I Universidade Federal da Bahia. Programa de Pós-Graduação em Saúde Coletiva. Salvador, BA, Brasil Universidade Federal da Bahia Programa de Pós-Graduação em Saúde Coletiva Salvador BA Brasil; II Universidade do Estado da Bahia. Departamento de Ciências da Vida. Salvador, BA, Brasil Universidade do Estado da Bahia Departamento de Ciências da Vida Salvador BA Brasil; III Universidade Federal da Bahia. Instituto de Psicologia. Salvador, BA, Brasil Universidade Federal da Bahia Instituto de Psicologia Salvador BA Brasil; IV Universidade Federal da Bahia. Instituto de Saúde Coletiva. Salvador, BA, Brasil Universidade Federal da Bahia Instituto de Saúde Coletiva Salvador BA Brasil; V Universidade Federal da Bahia. Instituto de Humanidades, Artes e Ciências Professor Milton Santos. Salvador, BA, Brasil Universidade Federal da Bahia Instituto de Humanidades, Artes e Ciências Professor Milton Santos Salvador BA Brasil

**Keywords:** HIV, PrEP, Indetectável, Jovens, Biotecnologias

## Abstract

**OBJETIVO:**

Neste artigo, discutimos como a profilaxia pré-exposição (PrEP) e a carga viral indetectável=intransmissível (CVI=I) têm produzido reconfigurações nos contextos de encontros afetivo-sexuais de jovens gays/ homens que fazem sexo com homens (HSH) vivendo com HIV (JVHIV).

**MÉTODOS:**

Conduzimos entrevistas em profundidade com nove JVHIV, de 18 a 29 anos, oriundos de duas pesquisas realizadas em Salvador/Bahia, em 2019 e 2021. Focalizamos narrativas sobre acontecimentos inéditos na prevenção e no tratamento do HIV/aids, que têm permitido experiências de maior intimidade e segurança, mas também desafios e tensionamentos às relações afetivo-sexuais.

**RESULTADOS:**

Momentos distintos da experiência de viver com HIV revelam diferentes narrativas dos JVHIV com relação às novas biotecnologias de PrEP e CVI. Preocupações em torno de uma possível transmissão do HIV ou da obrigação de revelar a sorologia ficam mais destacadas entre jovens com o diagnóstico mais recente, enquanto aqueles com maior tempo de sorologia se colocam de forma mais confortável e confiante frente às novas tecnologias e seus efeitos significativos nos encontros sexuais, ainda que permaneçam controvérsias a respeito das consequências morais e comportamentais do uso delas. Alguns JVHIV reatualizam preocupações e trazem relatos sobre a continuidade do estigma em relação às pessoas vivendo com HIV. Outros JVHIV enfatizam os benefícios dos avanços biomédicos, com abertura para novas possibilidades interativas, inclusive sem uso da camisinha, sublinhando a existência de outras práticas, saberes, dinâmicas e formas de negociação do risco/cuidado, com tensionamentos no campo da própria sexualidade.

**CONCLUSÕES:**

Reiteramos a necessidade de retomada de políticas públicas no campo do HIV/aids para além das estratégias biomédicas, dando destaque às vulnerabilidades, à disseminação de informações sobre as novas tecnologias de prevenção e tratamento do HIV, ao respeito à autonomia das pessoas em suas escolhas preventivas e ao desenvolvimento de estratégias de enfrentamento aos estigmas associados ao HIV/aids.

## INTRODUÇÃO

A ampliação do uso de medicamentos antirretrovirais (ARV) para prevenção do HIV, através das profilaxias pré e pós-exposição (PrEP e PEP, respectivamente) e do tratamento como prevenção (TcP), é resposta eficaz ao enfrentamento da epidemia do HIV/aids. Ao passo que produz impacto na prevenção de novas infecções, o uso dessas tecnologias farmacológicas traz benefícios às relações afetivo-sexuais e levanta desafios e novas questões para o cotidiano dos serviços de saúde e das pessoas^1^^), (^^2^.

As evidências sobre o TcP, produzidas por diversos estudos ao redor do mundo, são cada vez mais robustas ao argumentar que pessoas vivendo com HIV (PVHIV) e com carga viral indetectável (CVI) não transmitem o vírus em suas relações sexuais, o que se convencionou chamar de *indetectável igual a intransmissível* (I=I)^3^. Esse avanço biotecnológico tem produzido mais conforto na vida afetivo-sexual de PVHIV, representando um marcador de maior segurança e atenuando preocupações em torno de seus encontros sexuais^4^, embora persistam relatos sobre estigma, sentimentos de medo/culpa e receios de possível transmissão^2^.

Por outro lado, diferentes estudos demonstraram a eficácia da PrEP para evitar novas infecções por HIV^5^^), (^^6^^), (^^7^^), (^^8^. Na medida em que se adere consistentemente ao seu uso, a PrEP funciona como uma barreira química ao HIV, oferecendo alta proteção mesmo em relações sexuais sem a barreira física dos preservativos. Desse modo, a prevenção ganhou uma nova faceta, sublinhada pelo uso de ARV por pessoas que não vivem com o vírus. Tal aspecto tem conferido a elas um novo nível de agência, controle e maximização do prazer sensorial, permitindo que experimentem uma intimidade sem medo da infecção por HIV, ainda que continue existindo o risco para outras infecções sexualmente transmissíveis (IST).

Nesse contexto do sexo farmacologicamente seguro, o uso de ARV tem redefinido não apenas a forma como algumas pessoas lidam com o HIV, mas com a própria sexualidade, a gestão de sua saúde sexual e a concepção convencional de risco. Em particular, o conceito de “farmacopoder” desenvolvido por Preciado^9^ nos oferece uma lente valiosa para compreender esse contexto de profunda interseção entre farmacologia, corporeidade e identidade. Ao dar continuidade ao exame do que Foucault^10^ denominou como “biopoder”, ou seja, as estratégias de poder que atuam sobre a vida em uma escala populacional, Preciado destaca como substâncias e medicamentos têm desempenhado um papel crucial nas experiências de saúde/doença, produzindo nossas percepções sobre nós mesmos e nossa relação com os outros.

Desse modo, a concepção de “sexo protegido” tem sido redefinida, pois suas fronteiras agora estão borradas pela emergência dessas novas tecnologias, dado que a prevenção não se dá exclusivamente pelo preservativo, mas passa a ser interiorizada também no próprio corpo, por meio da ingestão de substâncias químicas^11^. Em contrapartida, uma vez que a PrEP e o I=I destacam-se por proporcionar entre seus usuários uma experiência sexual mais prazerosa e a redução da ansiedade relacionada à transmissão do HIV^12^^), (^^13^^), (^^14^, existem receios entre profissionais de saúde e mesmo entre aqueles que utilizam essas tecnologias sobre a possibilidade de maior engajamento em múltiplas parcerias sexuais, infidelidade, maior frequência de relações sexuais e redução ou mesmo abandono do preservativo^15^^), (^^16^^), (^^17^^), (^^18^. Simultaneamente, persiste a reprodução de imagens negativas em relação aos homens cis gays/homossexuais, tidos como sujeitos “excessivos”, ou, como problematiza Kane Race^19^, um certo “pavor” da retomada do sexo “sem freios” entre homens.

Nessa direção, buscamos compreender como essas novas tecnologias de prevenção e tratamento para o HIV têm mediado ou reconfigurado o sexo entre homens cis gays jovens vivendo com HIV (JVHIV). Mais precisamente, destacamos como esse aspecto aparece no quadro das distintas temporalidades do diagnóstico desses jovens, considerando tanto o período mais recente de vivência com a sorologia quanto aquele de maior tempo. Dessa forma, a partir da perspectiva de JVHIV, daremos destaque tanto às mudanças quanto às ambivalências e tensões que persistem nos encontros afetivo-sexuais, especialmente desconfianças e controvérsias emergentes a partir da introdução das tecnologias farmacológicas, como a PrEP e a CVI=I.

## MÉTODOS

Partimos de duas pesquisas distintas (PrEP15-19 e Sociabilidades Positivas (S+)), realizadas em Salvador/Bahia. Conduzimos entrevistas em profundidade com nove jovens gays/homens que fazem sexo com homens (HSH) vivendo com HIV, a partir de um roteiro semiestruturado, que abordou temas como rotinas de tratamento e cuidado, engajamento em relações afetivo-sexuais e suas perspectivas pós-diagnóstico de HIV.

A primeira rodada de entrevistas ocorreu no final do ano de 2019, entre os meses de outubro e dezembro, com quatro jovens de 18 e 19 anos, no decurso do estudo PrEP15-19. Mais informações metodológicas sobre esse estudo podem ser identificadas em Dourado et al.^20^ e Magno et al.^21^. Esses jovens foram diagnosticados com HIV durante suas primeiras visitas à clínica de prevenção combinada, para onde se dirigiram inicialmente em busca da PrEP. Após o diagnóstico, receberam orientações sobre prevenção, cuidados com a saúde e, então, foram encaminhados para tratamento no SUS. Posteriormente, foram convidados por uma profissional de saúde da clínica para cederem entrevista à equipe de pesquisa qualitativa do estudo. As entrevistas foram realizadas em período de 10 dias a 3 meses após o diagnóstico. Esses jovens estavam em início do tratamento com ARV.

A segunda sequência de entrevistas ocorreu, aproximadamente, um ano após a primeira rodada, em janeiro de 2021, com outros cinco jovens com idades entre 22 e 29 anos. Esses jovens participaram da pesquisa S+, cuja equipe de pesquisadores vem acompanhando suas trajetórias desde 2016, ano de seus diagnósticos^2^. No momento da entrevista, esses jovens relataram estar com carga viral indetectável=intransmissível (CVI=I). Ao longo do texto, citaremos entre aspas os fragmentos das narrativas dos jovens entrevistados, seguidos por seus nomes fictícios, como parte do nosso compromisso ético com o anonimato.

As entrevistas foram conduzidas por uma equipe composta por pesquisadores/as com treinamento em pesquisa qualitativa no campo da saúde. Foram realizadas individualmente, de forma presencial, em uma sala privativa nas dependências da clínica do estudo PrEP15-19. Ainda que produzidos no âmbito de dois estudos distintos, optamos por reunir o conjunto de materiais de ambas as pesquisas, por entendermos que delineiam aspectos dialógicos sobre as trajetórias afetivo-sexuais dos jovens participantes.

A análise dos materiais produzidos foi iniciada por meio de leituras exploratórias, a fim de identificar temas e questões que se deslocavam nas narrativas em torno da condição sorológica e dos contextos de encontros afetivo-sexuais desses jovens. Posteriormente, qualificamos os aspectos comuns dessas narrativas, com destaque para os novos acontecimentos na epidemia de HIV/aids, como o advento da PrEP e da CVI=I. Assim, ao longo da análise, discutiremos três aspectos ou temas evidenciados pelos materiais: (1) o desencontro entre avanços fármaco-médicos, que demarcam maior segurança em relação à infecção/transmissão do HIV, e a experiência cotidiana da sorologia ainda marcada pelo estigma e pelo medo da rejeição; (2) a reconfiguração dos modos como JVHIV lidam com a sorologia e as situações cotidianas de encontros afetivo-sexuais, especialmente ao longo do tempo de vivência com o HIV e com o acesso às evidências sobre sua condição de saúde; e, por fim, (3) a reprodução de velhos dilemas morais ligados ao exercício das sexualidades.

Compreendemos que as narrativas “pessoais” desses JVHIV se conectam ao enredo mais generalizado sobre a atual epidemia de HIV/aids, ao passo que reconhecemos que diferentes histórias são reatualizadas, emergem e coexistem em relação a esse enredo^22^. Portanto, ao longo da análise, consideramos a importância e a força das narrativas, com sua heterogeneidade e polifonia, operando a abertura de novas questões para esse campo de estudos^23^.

Sobre a caracterização dos participantes, sete dos entrevistados se identificaram como negros (pretos e pardos) e dois como brancos (Quadro 1). No momento da entrevista, todos os quatro participantes do estudo PrEP15-19 informaram residir com seus pais. Entre os cinco participantes da pesquisa S+, dois referiram residir com os pais, dois sozinhos e um dividia moradia com amigos. No que se refere à caracterização clínica, todos apresentavam quadro assintomático no momento da entrevista.

**Quadro 1:** Caracterização dos participantes

Nome fictício	Idade^a^	Orientação sexual	Raça/cor	Escolaridade	Relacionamento	Tempo de diagnóstico do HIV^a^
**Estudo PrEP15-19**						
Caio	18	Gay	Pardo	Ensino Médio incompleto	Solteiro	10 dias
Ruan	18	Gay	Pardo	Ensino Médio incompleto	Solteiro	1 meses
Henrique	19	Bissexual	Preto	Ensino Médio incompleto	Solteiro	3 meses
Eduardo	19	Gay	Pardo	Ensino Médio incompleto	Solteiro	5 meses
**Pesquisa Sociabilidades Positivas**						
Samuel	22	Gay	Preto	Superior incompleto	Solteiro	4 anos
Bernardo	27	Gay	Pardo	Superior completo	Solteiro	4 anos
Caetano	29	Gay	Branco	Superior completo	Namorando	4 anos
Douglas	27	Gay	Branco	Ensino Médio completo	Namorando	4 anos
Ramon	24	Gay	Pardo	Ensino Médio completo	Solteiro	4 anos

Fonte: Elaborado pelos autores. ^a^ No momento da entrevista.

Tomando como referência suas idades no momento da entrevista, sublinhamos que esses jovens tinham entre 18 e 24 anos quando diagnosticados, evidenciando que se trata de infecções ocorridas no início de suas experiências sexuais. Esse elemento apoia os dados apresentados pelos Boletins Epidemiológicos dos últimos anos, que vêm demonstrando o recrudescimento da epidemia no segmento etário juvenil e a necessidade de ampliação do debate sobre as suscetibilidades socialmente configuradas, que produzem vulnerabilidades ao HIV/aids, especialmente pela disponibilidade desigual de recursos para as pessoas se protegerem e para produção de saúde/cuidado.

A pesquisa Sociabilidades Positivas foi aprovada pelo Comitê de Ética em Pesquisa (CEP) do Instituto de Saúde Coletiva da Universidade Federal da Bahia (ISC/UFBA) (nº 1.684.862/2016). O estudo PrEP15-19 foi aprovado pelos CEP da Organização Mundial da Saúde (Identificação: Fiotec-PrEP Adolescent study) e do ISC/UFBA (nº 3.224.384). As entrevistas foram realizadas seguindo as diretrizes das resoluções 466/2012 e 510/2016, do Conselho Nacional de Saúde, sobre ética na pesquisa em ciências humanas e sociais em saúde.

## RESULTADOS E DISCUSSÃO

Na entrada da quinta década da epidemia do HIV/aids, centrada no progresso do tratamento e da prevenção fármaco-médica, novos arranjos relacionais despontam a partir do uso das fármaco-tecnologias. Nessa direção, destacam-se: relacionamentos afetivo-sexuais entre pessoas sorodiferentes, em que o status indetectável de um dos parceiros é um marcador de prevenção no interior da relação; ou relações em que um parceiro está indetectável e o outro em uso de PrEP; ou mesmo um sentido de companheirismo entre “semelhantes”, caracterizado pela “preferência” de PVHIV por se relacionar com parceiros de mesmo status sorológico, afastando, assim, determinados dilemas, tensões e questionamentos sobre sua sorologia^2^.

Ao tempo desses ineditismos na história da epidemia, ainda persistem entre PVHIV desafios ao considerar se devem revelar sua sorologia aos parceiros, por receio de serem estigmatizadas e rejeitadas e, por seu turno, o sentido de responsabilidade pessoal em evitar uma eventual infecção/transmissão do vírus. Tais aspectos estão tangenciados, por exemplo, pela ideia de que o HIV é uma condição da qual se deve ter vergonha, por estar especialmente vinculada às práticas sexuais “desviantes”. Esse quadro permitiu que Treichler^24^ cunhasse, desde a primeira década da epidemia, a expressão “epidemia de significados”, que se tornou clássica ao analisar como a linguagem - não apenas médica e científica - produz aquilo que concebemos como HIV/aids, fornecendo as bases para a produção de estigmas e seu efeito prático, que é a discriminação.

Neste estudo, argumentamos que esse quadro é atualizado diante de outros atores/actantes, como a CVI=I e a PrEP^18^, que, fazendo parte de uma rede de interações, têm contribuído para reconfigurar ou dar contornos inéditos não apenas à clínica da prevenção e do tratamento, mas também aos relacionamentos afetivo-sexuais. É o caso, por exemplo, de se “viver com HIV” e, no entanto, afirmar-se como “negativo usando PrEP”, conforme destacado por um dos nossos interlocutores, com CVI=I, a partir de suas incursões em aplicativos de relacionamento:

Eu vejo muita gente que tem HIV/aids e diz que tá usando PrEP. Vejo isso em aplicativos, conversas, grupos. Num grupo mesmo, disseram que alguém tinha aids e ele falou que tomava PrEP e era negativo. Eu conversei com um menino que me falou que era HIV+ indetectável; eu queria dar um vácuo, mas não podia, aí falei que era negativo usando PrEP. (Bernardo, 27 anos)

Essa narrativa reitera como a experiência cotidiana da sorologia parece ainda estar em descompasso em relação aos avanços fármaco-médicos ocorridos nos últimos anos da epidemia. Ou seja, ainda que esses novos avanços possam borrar a fronteira entre “negativos” e “positivos”, ou melhor, entre “ausência” e “presença” do vírus HIV^25^, considerando aqui a categoria indetectável, há de se destacar a persistência do medo ou receio de ser associado ao HIV/aids, com a consequente discriminação. Nesse sentido, para além da medicalização e normalização do HIV^26^^), (^^27^ como uma doença crônica e tratável, não se pode esquecer dificuldades e dilemas que persistem neste momento histórico que Simões denomina como “nova aids”^28^, resultante de exitosas intervenções biomédicas sobre sua realidade clínica e epidemiológica.

Vale enfatizar que diferentes estudiosos/as do tema^29^^), (^^30^^), (^^31^ têm reiterado que, atualmente, há uma ênfase em perspectivas individuais no enfrentamento à epidemia do HIV/aids, com o relativo deslocamento da prevenção dos espaços comunitários/coletivos para o interior da clínica médica, especialmente centradas no ato de medicar. Nessa direção, um conjunto de novos saberes sobre o HIV e as novas tecnologias, como I=I e PrEP, não têm circulado para além de nichos específicos, contribuindo para manter o caráter persistente do estigma. Apesar disso, conforme é discutido neste artigo, há também mudanças importantes possibilitadas por essas novas biotecnologias. Nas seções que seguem, alguns desses aspectos do viver com HIV e das relações afetivo-sexuais ganham diferentes contornos a partir de distintas temporalidades.

### O tempo da descoberta sorológica: rupturas e tensões em foco

A sorologia positiva para HIV continua enredando a produção de dilemas e sentidos de culpa, individualizando, simplificando e minimizando outros elementos em torno dessa condição de saúde^32^^), (^^15^. Como ocorre com outras doenças crônicas, em relação às rupturas biográficas^33^ - mas principalmente pelo estigma do HIV, vinculado à ideia de “sexualidades promíscuas”^26^ e “corpos perigosos”^34^^), (^^35^ -, esse novo diagnóstico produz também questionamentos sobre os próprios sujeitos e seus modos de vida^15^. Assim, o reengajamento em relações pode implicar dificuldades, especialmente no início da vivência da sorologia, como pode ser exemplificado por relatos de alguns de nossos interlocutores com tempo mais recente de diagnóstico.

Na primeira vez que eu fui fazer [sexo] depois do diagnóstico, eu fiquei com medo. Mas tentei me tranquilizar, tentei não pensar. Teve alguns momentos que eu pensei na questão do HIV e tudo mais, com medo de acontecer tanta coisa, da camisinha estourar e acontecer isso ou aquilo. [...] Me sinto um pouco assim... eu só fico analisando cada passo, porque qualquer coisa que a pessoa fizer pode acabar me afetando e eu vou ter que acabar contando [que tenho HIV] pra pessoa, entendeu?. (Caio, 18 anos)

No que diz respeito ao dilema da revelação sorológica - “ter que acabar contando” -, Agostini et al.^36^ enfatizaram em seu trabalho como a administração da condição de soropositividade apresenta desafios no contexto dos relacionamentos afetivo-sexuais, incluindo a necessidade de estabelecer um vínculo de confiança antes que o tema da sorologia seja abordado. Assim, a gestão do segredo desempenha um papel importante nessas relações, sendo mantida como estratégia de proteção contra o estigma, a rejeição afetiva e a discriminação. Os autores também discutem como alguns desses jovens demonstram uma grande preocupação em evitar a transmissão e adotam estratégias de prevenção, como o preservativo.

Ainda que tensões relacionadas ao envolvimento sexual não se limitem ao período inicial de vivência da sorologia, as narrativas dos interlocutores do estudo PrEP15-19 - conforme relatado acima, por Caio - enfatizam mais esse aspecto do que as narrativas dos jovens da pesquisa S+. Sobre isso, a partir de uma perspectiva de construção de sentidos ao longo do tempo, o estudo de Anjos^37^ observou que o choque inicial do diagnóstico, frequentemente, progride para uma aceitação gradual dessa condição por JVHIV. O autor explorou como a vivência com o HIV é permeada por um período inicial de ajustamento emocional e dificuldades na revelação do status sorológico aos familiares e parceiros, devido à preocupação de serem estigmatizados e excluídos. No entanto, destaca a resiliência e a busca por uma vida significativa por parte desses jovens, apesar dos desafios, medos e incertezas presentes na vivência com o HIV. Ele também enfatiza como a temporalidade do diagnóstico, seja ela recente ou mais longa, cumpre papel significativo na forma como JVHIV convivem com sua sorologia, se adaptam a essa condição de saúde e lidam com seus encontros afetivo-sexuais.

Embora seja importante reconhecer que o estigma, o medo e a discriminação ainda sejam obstáculos significativos, independentemente do tempo desde o diagnóstico, esses estudos sinalizam que aqueles que vivem com HIV por um período mais longo, com frequência, têm uma melhor compreensão sobre sua condição de saúde, desenvolvem estratégias de enfrentamento resilientes, aprendem a gerenciar aspectos práticos e emocionais da sorologia e estabelecem um senso mais estável de identidade positiva em relação à sua soropositividade.

Nossos interlocutores recém-diagnosticados e ainda no início do tratamento destacaram histórias sobre como o envolvimento sexual pós-diagnóstico está rodeado por tensões, que podem significar uma frequência maior de preocupações que antes do diagnóstico pareciam menos pregnantes, como o uso do preservativo. Esses cuidados se relacionam com os novos sentidos de risco ou “ameaça” para o outro que o diagnóstico de HIV passa a mobilizar na vida desses jovens, a camisinha começa a ser vista como algo compulsório ou mesmo como uma obrigação moral.

Eu uso camisinha pra tudo agora, camisinha pra tudo. Sem contar que... eu to tendo muito mais cuidado. Mesmo com camisinha, eu acho bacana você aprofundar um pouco mais antes de fazer qualquer coisa. Principalmente a parte de você usar camisinha pra tudo, foi depois do projeto [PrEP15-19, no qual foi diagnosticado]. Isso eu não fazia, não. (Ruan, 19 anos)

Hoje em dia eu sempre uso [preservativo], ele querendo ou não. Se não quiser, não transo. Antes eu não tinha isso. Se quisesse, usava; se não quisesse, normal [não usava]. (Eduardo, 19 anos)

Esses elementos permanecem ao longo da experiência da sorologia, implicando desafios significativos para JVHIV. No entanto, em momentos distintos de suas trajetórias outras preocupações vêm à tona, as quais ainda dizem respeito à segurança, mas que introduzem novos sentidos para as relações sexuais, como liberdade e prazer.

### A PrEP e CVI=I em cena: novas possibilidades interativas, de negociação do sexo e seus tensionamentos

No contexto de segurança farmacológica do sexo, estratégias como a PrEP e a CVI=I têm potencializado o bem-estar sexual de PVHIV^38^. Destacamos as histórias de encontros sexuais relatados por Caetano - 29 anos e vivendo com HIV desde os 24 anos - e Douglas - 27 anos e vivendo com HIV desde os 25 anos. São contextos em que o uso da camisinha é contingencial a elementos como “despreocupação”, ”conforto”, “estabilidade”, “confiança”, “negociação” e “prazer”.

Quando estávamos marcando [um encontro], ele deixou claro que a minha sorologia não fazia diferença pra ele e que ele usava PrEP. [...] Acho que é a primeira vez que eu transo sem camisinha, sem ficar muito preocupado com o outro. [...] Agora eu estou tendo o prazer de transar com alguém que faz uso da PrEP e algumas vezes transamos sem camisinha. Essa zona de conforto em relação à proteção faz a gente desfrutar mais. (Caetano, 29 anos).

[...] Hoje em dia a gente faz mais sem camisinha do que antigamente. [...]. Agora que eu estou indetectável, ele parou [de tomar PrEP]. Ele até descobriu uma forma diferente de usar a PrEP, usa um dia, depois dois dias, depois mais dois. (Douglas, 27 anos).

Por meio desses fragmentos, enfatizamos aspectos que passam a marcar o contexto atual do HIV/aids, ou seja, a referência às novas biotecnologias como elementos que fazem a diferença na tomada de decisão no interior das relações afetivo-sexuais. As narrativas de JVHIV que alcançaram a indetectabilidade viral apontam elementos que marcam a experiência da sorologia hoje e oferecem uma nova dinâmica no que diz respeito à segurança. Nesse caso, a indetectabilidade produz uma sensação de liberdade na exploração do prazer sexual e mudanças no modo como esses JVHIV desfrutam de relações sem preocupações excessivas com a transmissão do vírus, ao mesmo tempo em que enfrentam desafios envolvendo a comunicação e a negociação com parceiros. Em consonância com as reflexões de Preciado^9^, esse contexto ressalta o cruzamento entre farmacologia, corporeidade e identidade, no qual a medicina e a tecnologia exercem papeis fundamentais na redefinição das experiências sexuais e na construção de novos sentidos para as relações íntimas.

As experiências relatadas por esses e outros jovens demonstram como, a partir da disponibilidade e da disposição para usar outras tecnologias que não o preservativo, as pessoas podem combinar e negociar usos, colocando na balança preocupações e interesses sexuais na medida em que desejam experimentar o prazer de forma “mais livre” e “sem barreiras”. Assim como sublinhado por Silva et al^18^, tais fragmentos evidenciam como a prevenção pode ser ajustada ou reconfigurada conforme a atuação de tecnologias (I=I, PrEP, preservativo), o tipo de relacionamento (fixo, casual, aberto, fechado), o tempo do diagnóstico ou mesmo diante de situações em que a relação risco-prazer é dialogada entre os parceiros.

Entretanto, é preciso destacar que, para além das evidências de benefícios e da efetividade da PrEP e da CVI=I como prevenção ao HIV, entre alguns desses jovens também se apresentam preocupações sobre as consequências morais e comportamentais da disponibilidade desses novos recursos preventivos. Essas preocupações estão especialmente vinculadas à imagem negativa de “promiscuidade” atribuída aos homens gays^39^. Como veremos adiante, alguns dos nossos interlocutores percebem o sexo sem preservativo, mesmo entre aqueles que estão em uso de PrEP, como uma ação desinformada ou algo “banalizado”. Além disso, no plano simbólico, a associação da PrEP à “promiscuidade” e à “irresponsabilidade” pode conduzir à estigmatização das pessoas que aderem ao método, principalmente quando este é reputado como uma estratégia ligada à “loucura”, à “desproteção” e ao “descuido”.

Eu acho a PrEP importante, mas eu acho que rolou uma banalização da ideia de segurança depois da PrEP. A PrEP é importante, mas a camisinha é indispensável [...], mas aí, algumas pessoas acham que, por estarem tomando a PrEP, estão protegidas de tudo. Aí nos aplicativos de pegação você vê que as pessoas só se confiam na PrEP. (Samuel, 22 anos).

Muita gente está confiando na PrEP, mas a PrEP não protege outras doenças. [...] É que as vezes a pessoa, quando tá namorando, pode se sentir segura em fazer sexo sem camisinha. Tem gente que é louca. Tem amigos meus que fazem sexo sem camisinha quando estão namorando. Eu fico muito em aplicativo e vejo muita gente querendo *barebacking* pra cima e pra baixo. As pessoas confiam muito numa pílula que pode falhar, no caso, a PrEP. Eu já vi documentários de pessoas que tomavam PrEP e mesmo assim pegaram HIV. [...] Se for uma pessoa desleixada, não dá certo. (Bernardo, 27 anos)

Ao discutirem confiança na eficácia do medicamento, esses jovens revelam suas preocupações quanto ao uso da PrEP associadas aos riscos de outras IST e à imagem negativa de “promiscuidade” gay em aplicativos de encontro. Suas suspeições dizem respeito aos riscos que nesse caso são vistos como negativos e, portanto, como algo a ser evitado. Como destaca Lupton^40^, esta ênfase em evitar o risco está fortemente associada a um desejo de controle e racionalização da vida e do corpo, evitando as “vicissitudes do destino”. Nesse cenário, o sexo sem preservativo, mesmo entre aqueles que estão indetectáveis ou em uso de PrEP, pode ganhar contornos de “imprudência” e “desconfiança”, sendo concebido mais como um sexo malcomportado, susceptível à “falha”, ao “descontrole” ou à “desrazão/loucura”, do que como um “ponto de equilíbrio” entre custos e benefícios do risco e do comportamento preventivo, como ressaltado por Eaton e Kalichman^41^, ou mesmo como parte de uma rede complexa de negociação de perdas e ganhos^42^, em que se pondera o medo/risco frente aos interesses sexuais e à busca por prazer^14^.

Para Spink^43^, embora continue anunciando a possibilidade da perda (“pegar HIV/IST”), o risco também pode ser vivido como uma experiência positiva (negociação, intimidade, prazer), uma vez que é possível afrontar os limites, “calibrar” prudência e desejo, perda e recompensa, perigo e prazer, risco e segurança, rompendo com uma racionalidade preventiva que desconsidera novos recursos e tecnologias que incidem sobre o desejo, a intimidade e a segurança. A PrEP e a CVI=I acontecem em meio a essas controvérsias e tensionamentos no cotidiano das pessoas, especialmente porque é de fato nessas interações, nas práticas e nas situações concretas que essas tecnologias passam a existir.

## CONSIDERAÇÕES FINAIS

Neste artigo, abordamos narrativas de JVHIV, dando destaque a alguns aspectos que persistem na vivência soropositiva, principalmente no contexto das suas parcerias sexuais. Embora tenhamos evidenciado conflitos morais que persistem em relação ao HIV, nossas descobertas apontam para um cenário marcado pela reconfiguração das experiências afetivo-sexuais de JVHIV pelas novas tecnologias farmacológicas de CVI=I e PrEP, que produzem maior sentido de segurança e prazer. Por sua vez, a confiança na atuação dos medicamentos ARV como uma barreira química à transmissão/infecção por HIV também levanta questões sobre outros aspectos da prevenção, como acesso, equidade, cidadania e direito à saúde.

Certamente, novos estudos serão necessários para acompanhar possíveis mudanças nesse cenário, considerando os momentos distintos do viver com HIV, outras trajetórias de vida e novas tecnologias que venham a surgir. Neste artigo, por exemplo, preocupações em torno de uma possível transmissão do HIV ou da obrigação de revelar a sorologia ficam mais destacadas entre jovens com o diagnóstico mais recente, enquanto aqueles com maior tempo de sorologia se colocam de forma mais confortável e confiante frente às novas tecnologias e seus efeitos significativos nos encontros sexuais, na medida em que esses medicamentos remodelam as experiências sobre si mesmos e na relação com os outros, ainda que controvérsias insistam em se apresentar.

Nessa direção, destaca-se também a necessidade de retomada de políticas públicas no campo do HIV/aids que articulem as evidências sobre novas tecnologias de prevenção e tratamento às experiências de mobilização das quais as comunidades mais afetadas pela epidemia são protagonistas. Essas políticas devem estar ancoradas no horizonte dos direitos humanos e, por sua vez, ser culturalmente sensíveis, considerando as diferenças geracionais, os distintos pertencimentos sociais e o respeito à autonomia das escolhas preventivas e de cuidado. Além disso, devem desenvolver estratégias de redução de vulnerabilidades a partir do enfrentamento de contextos materiais, culturais e políticos de injustiça social e discriminação às pessoas e às comunidades afetadas pela epidemia^29^.

Finalmente, como Mol^44^ nos informa, o cuidado é múltiplo, pois se faz nas práticas, com suas imprevisibilidades, diferentes relações e condições, podendo ocorrer novas experimentações, com o objetivo de se produzir uma existência possível. No campo da prevenção, esse aspecto é relevante na medida em que é preciso considerar a existência de outras práticas, saberes, dinâmicas e formas de negociação do risco/cuidado, levando-se em consideração as dimensões do desejo/prazer e os tensionamentos no campo da própria sexualidade, como uma “zona fronteiriça” de coexistência diversa, por exemplo, de norma e transgressão^45^.

Nessa direção, a PrEP e a CVI=I levantam desafios, mas também oportunidades para os serviços de saúde, profissionais e usuários no que se refere ao enfrentamento de tabus relacionados à sexualidade e ao HIV/IST. Ademais, elas provocam a necessidade de se produzir novas abordagens de prevenção e cuidado que considerem as vulnerabilidades e os processos de estigmatização ou de desvalorização do outro.
